# Matters arising: methodological issues on evaluating agreement between medical students’ attitudes towards drugs/alcohol use during pregnancy by Cohen’s kappa analysis

**DOI:** 10.1186/s12909-023-04071-0

**Published:** 2023-02-17

**Authors:** Tianfei Yu, Lei Yang, Xinjie Jiang, Shuli Shao, Wei Sha, Ming Li

**Affiliations:** 1 College of Life Science and Agriculture Forestry, Qiqihar University, Qiqihar, 161006 China; 2 College of Foreign Languages, Qiqihar University, Qiqihar, 161006 China; 3 College of Computer and Control Engineering, Qiqihar University, Qiqihar, 161006 China

**Keywords:** Substance use, Pregnancy, Medical students, Attitudes, Agreement, Cohen’s kappa, Weighted kappa

## Abstract

**Background:**

The purpose of this article is to discuss the statistical methods for agreement analysis used in Richelle’s article (BMC Med Educ 22:335, 2022). The authors investigated the attitudes of final-year medical students regarding substance use during pregnancy and identified the factors that influence these attitudes.

**Methods:**

We found that Cohen’s kappa value for measuring the agreement between these medical students’ attitudes towards drugs/alcohol use during pregnancy was questionable. In addition, we recommend using weighted kappa instead of Cohen’s kappa for agreement analysis at the presence of three categories.

**Results:**

The agreement improved from “good” (Cohen’s kappa) to “very good” (weighted kappa) for medical students’ attitudes towards drugs/alcohol use during pregnancy.

**Conclusion:**

To conclude, we recognize that this does not significantly alter the conclusions of the Richelle et al. paper, but it is necessary to ensure that the appropriate statistical tools are used.

## Background

We read with interest the article entitled: “Factors influencing medical students’ attitudes towards substance use during pregnancy” which was published in *BMC Medical Education* on 2 May 2022 [1]. The authors investigated the attitudes of final-year medical students regarding substance use during pregnancy and identified the factors that influence these attitudes. They focused on two items, including drugs and alcohol, regarding the punishment of substance use during pregnancy. Nonetheless, we found that Cohen’s kappa value for measuring the agreement between these medical students’ attitudes towards drugs/alcohol use during pregnancy was questionable. We recommend using weighted kappa instead of Cohen’s kappa for agreement analysis at the presence of three categories. The agreement improved from “good” (Cohen’s kappa) to “very good” (weighted kappa) for medical students’ attitudes towards drugs/alcohol use during pregnancy. To conclude, we recognize that this does not significantly alter the conclusions of the Richelle et al. paper, but it is necessary to ensure that the appropriate statistical tools are used.

## Main text

Cohen’s kappa statistic is generally suitable for evaluating two raters [2]. Especially in Cohen’s kappa statistic, the weighted kappa statistic should be used to estimate the inter-rater reliability in the presence of more than two categories [3]. In contrast to Cohen’s kappa statistic, weighted kappa statistic relies more on predefined cell weights that reflect agreement or disagreement.

Cohen’s kappa is calculated as follows:

1 $${k}_C=\frac{\sum_{j=1}^n{u}_{jj}\left(i{i}^{\prime}\right)-\sum_{j=1}^n{p}_{ij}{p}_{i^{\prime }j}}{1-\sum_{j=1}^n{p}_{ij}{p}_{i^{\prime }j}}$$

Weighted kappa is calculated as follows:

2 $${k}_w=1-\frac{\sum_{i=1}^n\sum_{j=1}^n{w}_{ij}{p}_{ij}}{\sum_{i=1}^n\sum_{j=1}^n{w}_{ij}{p}_i{q}_j}$$

The value of *u*_*jj*_(*ii*^′^) is the proportion of objects put in the same category *j* by both raters *i* and *i*^′^. The value of *p*_*ij*_ is the proportion of objects that rater *i* assigned to category *j*, and *k* is the number of raters. Cohen [4] suggested the *k* value should be interpreted as follows: < 0.20 as poor agreement, 0.20–0.40 as fair agreement, 0.41–0.60 as moderate agreement, 0.61–0.80 as good agreement, and > 0.80 as very good agreement.

In the authors’ Table 1, according to the authors’ calculation, inter-rater reliability was good for medical students’ attitudes towards drugs/alcohol use during pregnancy (Cohen’s kappa = 0.775, 95% [confidence interval] CI = 0.714–0.837). But, in our opinion, weighted kappa was more applicable than Cohen’s kappa for the presence of three categories. Consequently, we performed the weighted kappa statistics (linear and quadratic) for evaluating the agreement according to the authors’ data. The linear weighted kappa value was 0.804 (95% [confidence interval] CI = 0.746–0.863) as very good agreement. The quadratic weighted kappa value was 0.831 (95% [confidence interval] CI = 0.770–0.892) as very good agreement, too. The greater the difference between the ratings of the same data, the stronger the hint of inconsistency. For example, the penalty for category disagree into category agree should be significantly greater than that for predicting category disagree into category undecided. If using Cohen’s kappa, there is no difference between the former and the later. If using linear weights, the penalty for the former is equal to 2 times the later. If using quadratic weights, the penalty for the former equal to 4 times the later. Therefore, we recommend that quadratic weighted kappa should be used to evaluate the agreement for magnifying the degree of inconsistency in the judgment of large level distance.

**Table 1:** Punishment for pregnant women using drugs or alcohol

Drugs	Alcohol		
	Disagree	Undecided	Agree
Disagree	200	10	6
Undecided	12	57	11
Agree	13	1	50
*k*_*c*_: 0.775 (*p*<0.001, 95% [confidence interval] CI = 0.714–0.837)			
*k*_*lw*_: 0.804 (*p*<0.001, 95% [confidence interval] CI = 0.746–0.863)			
*k*_*qw*_: 0.831 (*p*<0.001, 95% [confidence interval] CI = 0.770–0.892)			

The data has been cited from the article published by Richelle et al. [1] and undergone modification. *k*_*c*_: Cohen’s kappa, *k*_*lw*_: linear weighted kappa, *k*_*qw*_: quadratic weighted kappa

In conclusion, the authors underestimated the agreement between medical students’ attitudes towards drugs/alcohol use during pregnancy. The reasonable agreement was “very good” for medical students’ attitudes towards drugs/alcohol use during pregnancy. Anyway, we recognize that this does not significantly alter the conclusions of the Richelle et al. paper, but it is necessary to ensure that the appropriate statistical tools are used. We highlight that the rigor and use of the correct statistical approach is crucial for any scientific publication. Applying appropriate statistical methods can enhance the scientific accuracy of research results.

## Data Availability

Not applicable.

## Abbreviation

CI: Confidence interval
